# Modelling and Simulation of Collective Cell Migration with Non-Local Interactions on Time-Dependent Spatial Domains

**DOI:** 10.1007/s11538-026-01628-0

**Published:** 2026-03-24

**Authors:** Alf Gerisch

**Affiliations:** Research Group Numerical Analysis and Scientific Computing, Department of Mathematics, Technische Universität Darmstadt, Dolivostr. 15, 64293 Darmstadt, Germany

**Keywords:** PDE model, Collective cell migration, Non-local interaction, Time-dependent domain, Numerical simulation, Computational complexity

## Abstract

We extend the formulation of a non-local PDE model of collective cell migration involving attracting or repelling cellular interactions to the case of time-dependent spatial domains as present, for instance, in modelling developmental processes from embryology. We restrict to a spatially one-dimensional setting, as is appropriate for the modelling of neural crest cell invasion, and focus on the case of spatially homogeneous domain change as this already highlights many of the modelling and numerical challenges. The approach is illustrated and numerical simulations are presented and discussed for a model of an aggregating cellular population and for a simple model of neural crest cell invasion accounting for contact inhibition of locomotion.

## Introduction

The collective migration of cells, driven by appropriate cell-cell and cell-tissue interactions, is fundamental in many biological systems with applications ranging from developmental biology (Scarpa and Mayor 2016) and wound healing (Grada et al. 2017) to cancer progression (Li et al. 2013) and many more, see also Friedl and Gilmour (2009) for a review.

Direct contacts^1^ between a cell and other cells or the underlying tissue are an important type of interaction in collective cell migration (Painter et al. 2015) and provide cues which instruct the cell to migrate. They can have an attracting, like in cellular adhesion, but also a repelling, like in contact-inhibition of locomotion, effect. These contacts and the associated exchange of information often take place not only in the direct vicinity of a cell but, via cellular protrusion, at distances multiple cell diameters away giving rise to so-called non-local models. The basic form of such a non-local model, in the context of a continuous modelling framework, has been introduced nearly twenty years ago by Armstrong et al. (2006) and is widely used and extended for various applications ever since (Painter et al. 2015, 2023; Buttenschön et al. 2017; Murakawa and Togashi 2015; Carrillo et al. 2019). A comprehensive discussion of non-local models is also available in the book (Buttenschön and Hillen 2021) and recent results on the existence and positivity of solutions for such non-local models are given in Giunta et al. (2022, 2024).

Looking at particular applications from developmental biology or from cancer progression, it is evident that such processes take place in spatial domains which change shape and size with time. Clearly, if the resulting *time-dependent spatial domains* have an impact on the process of collective cell migration then they must be included in the modelling. In the context of local models of reaction-diffusion-chemotaxis type this is, for instance, considered from a modelling as well as a numerical simulation point of view in Crampin et al. (1999, 2002); Landman et al. (2003); Giniūnaitė et al. (2020); Simpson et al. (2006). Madzvamuse and co-workers place a particular focus on the impact of domain shape and of time-dependent domains on the pattern forming potential of reaction-diffusion systems. Their numerical approach uses (moving) finite element approximations in space and, amongst others, dedicated implicit-explicit time-stepping schemes in time, see, *e.g.*, Madzvamuse (2000); Lakkis et al. (2013).

The modelling of collective cell migration is reviewed in Giniūnaitė et al. (2020) and discrete, individual-based, models as well as continuous, partial differential equation (PDE), models are considered. These authors use the process of neural crest (NC) cell migration as their main illustrating example. They emphasise the importance of domain growth as well as of non-local interactions for collective cell migration which give rise to so-called integro-PDE models on time-dependent spatial domains. Giniūnaitė et al. (2020) state in their Section 5 *that it is challenging to implement integro-PDE models on a growing domain due to changes in the domain of integration of the interaction terms [... and to] our knowledge there are no studies in this area*. Bridging this gap — by proposing a viable computational approach to the simulation of non-local PDE models on time-dependent spatial domains — in a simple yet instructive setting is one of the aims of this work here. Another outcome of this work here is, that having such a modelling framework and a corresponding simulation environment for PDE models of collective cell migration with non-local interactions on time-dependent spatial domains available, this will enable and stimulate the investigation of many specific biological processes with these characteristics. It will also enable to assess and quantify the impact of spatial domain change in such systems on e.g. their pattern forming potential.

The paper is organised as follows. We begin in §2 with a review of time-dependent spatial domains and conservation laws on them. We focus on spatially one-dimensional domains, and more details on them are also collected in Appendix A, and in that case the time-dependent spatial domain can easily be transformed to a fixed spatial domain. The conservation law transforms accordingly and this is also presented. The material is then used to formulate a general non-local model on a time-dependent one-dimensional spatial domain for the mass density of a single cellular population in §3. The model includes terms to account for cell random motility and cell proliferation as well as a non-local interaction term and is supplemented with periodic boundary conditions (BCs) and an initial condition (IC). This model is also given in its form after transformation to a fixed spatial domain. That form of the model is the starting point for the numerical scheme to compute approximate model solutions. We describe in §4 essential points of our numerical approach and the impact of the time-dependent spatial domain on its efficiency. We then proceed to specify two specific non-local models on a time-dependent spatial domain and present and discuss selected simulation results: a model of an aggregating cellular population in §5 and a model of NC cell migration in §6. In Appendix B we additionally present a Fourier-type stability analysis for the model of §5 on a fixed spatial domain of variable length. We conclude this paper by summarizing our main results as well as by discussing some future extensions of the current work in §7.

## Time-Dependent Domains and Conservation Laws

In this section we consider spatially *d*-dimensional time-dependent domains in §2.1 and conservation laws on them in §2.2. This is followed by a special consideration of the spatially one-dimensional case, which is the focus of this study, in §2.3. There we also discuss the transformation to a fixed spatial domain and the correspondingly transformed conservation law.

### Time-Dependent Domains

We consider a finite time interval $I:=[t_0,T]\subset \mathbb {R}$ with initial time $t_0$ and final time $T>t_0$. For each time point $t\in I$ we have a corresponding *d*-dimensional spatial domain denoted $\Omega _t\subset \mathbb {R}^{d}$. We denote the time and space variables by *t* and *x*, respectively. In short we can write the space-time domain as

$$ \Omega _I:=\{(t,x)\in \mathbb {R}^{d+1}\,:\, t\in I\,, x\in \Omega _t\}\subset \mathbb {R}^{d+1}\,. $$

We consider here the case that this domain is generated by a velocity function $\textbf{v}:\Omega _I\rightarrow \mathbb {R}^{d}$ through the following initial value problem (IVP) with parameter $x_0\in \Omega _{t_0}$ for a system of ordinary differential equations (ODEs)

1 $$\begin{aligned} \dot{x}(t;x_0) = \textbf{v}(t,x(t;x_0)) \text { for all } t\in (t_0,T] \text { and } x(t_0;x_0) = x_0\,. \end{aligned}$$

Here, $\dot{x}(t;x_0)$ denotes the temporal derivative of the trajectory $x(t;x_0)$ emanating from $x_0\in \Omega _{t_0}$. We assume that the smoothness of $\textbf{v}$ is such that (1) has a unique solution on *I* for any $x_0\in \Omega _{t_0}$. This is, for example, ensured if $\textbf{v}$ is continuous on $I\times \mathbb {R}^{d}$ and fulfils a Lipschitz condition with respect to its second argument there, *cf.* (Strehmel et al., 2012, Theorem 1.2.1).  The uniqueness of solutions in particular ensures that trajectories emanating from any two different points in $\Omega _{t_0}$ do not cross or touch. In a slight abuse of notation one can now write compactly $\Omega _t=x(t;\Omega _{t_0})$ for all $t\in I$ . We remark that although in this study we restrict to the case that $\textbf{v}$ is known *a priori*, it can also be a solution of a differential equation itself; we comment on that more general case in §7.

The Jacobian matrix $\mathcal {J}_{x}\textbf{v}(t,x)$ corresponds to the local rate (per unit time) of change in domain size. If that rate is independent of *x* then we speak about *spatially homogeneous domain change*, otherwise *spatially heterogeneous domain change*. As a starting point for more complex modelling situations, most of the content in this study is concerned with spatially homogeneous domain change.

### Conservation Laws

We now turn the attention to the formulation of conservation laws on time-dependent domains $\Omega _I$ and consider a conserved quantity described by function $u:\Omega _I\rightarrow \mathbb {R}$. For simplicity assume that *u* is a mass density of a cellular population or a chemical concentration. Let $\omega _t\subset \Omega _t$ be a subdomain which evolves in time in the same way as $\Omega _t$ does, that is according to the velocity function $\textbf{v}$. Then the total mass (of the cellular population or the chemical) in $\omega _t$ is given by $\int _{\omega _t} u(t,x)\,\textsf{d}{x}$ and its change over time as

2 $$\begin{aligned} \frac{\,\textsf{d} }{\,\textsf{d}{t}}\int _{\omega _t} u(t,x)\,\textsf{d}{x} = -\int _{\partial {\omega _t}}\textbf{F}\cdot \mathfrak {n}\,\textsf{d}{(\partial {\omega _t})} + \int _{\omega _t}f(t,x)\,\textsf{d}{x}\,, \end{aligned}$$

where, as in the fixed-domain case,

- the first term on the right-hand side accounts for mass exchange through the boundary $\partial \omega _t$ as given by the normal flux $\textbf{F}(t,x)\cdot \mathfrak {n}(x)$ where $\textbf{F}:\Omega _I\rightarrow \mathbb {R}^{d}$ is the flux vector function and $\mathfrak {n}(x)$ is the unit outward normal vector at $x\in \partial \omega _t$, and
- the second term on the right-hand side accounts for mass production/destruction inside of $\omega _t$ as given by the source density $f:\Omega _I\rightarrow \mathbb {R}$.

For *d*-dimensional spatial domains, SI-units of *u*, $\textbf{F}$ and *f* are thus $\text {kg}\cdot \text {m}^{-d}$, $\text {kg}\cdot \text {m}^{-(d-1)}\cdot \text {s}^{-1}$, and $\text {kg}\cdot \text {m}^{-d}\cdot \text {s}^{-1}$ and that of $\textbf{v}$ is $\text {m}\cdot \text {s}^{-1}$.

We now transform Eq. (2), working from left to right, such that we obtain a differential equation in the end. Firstly, and in contrast to the fixed-domain case, we cannot just exchange the time derivative and the integral on the left-hand side of Eq. (2) due to the time-dependent integration domain $\omega _t$. Instead we apply Reynolds transport theorem, see, *e.g.*, Chorin and Marsden (1993), resulting in

$$ \frac{\,\textsf{d} }{\,\textsf{d}{t}}\int _{\omega _t} u(t,x)\,\textsf{d}{x} = \int _{\omega _t}\left[ \partial _t u(t,x) +\nabla \cdot \left( u(t,x)\textbf{v}(t,x)\right) \right] \,\textsf{d}{x}\,. $$

Observe that this brings the velocity $\textbf{v}$, driving domain change, into the equation. Secondly, and this time as in the fixed-domain case, we apply the divergence theorem to the surface integral on the right-hand side of Eq. (2). Combining these two steps, Eq. (2) transforms, after bringing all terms to the left-hand side, to

3 $$\begin{aligned} \int _{\omega _t}\left[ \partial _t u(t,x) +\nabla \cdot \left( u(t,x)\textbf{v}(t,x)+\textbf{F}(t,x)\right) -f(t,x)\right] \,\textsf{d}{x}=0\,. \end{aligned}$$

Note that this holds for any $\omega _t\subset \Omega _t$ and any $t\in I$. Therefore, under suitable smoothness assumptions, *e.g.* continuity of the integrand of (3), it follows,

4 $$\begin{aligned} \partial _t u(t,x) =-\nabla \cdot \left( u(t,x)\textbf{v}(t,x)+\textbf{F}(t,x)\right) +f(t,x)\quad \text {for all }x\in \Omega _t\,, t\in (t_0,T]\,. \end{aligned}$$

This (differential form of the) equation of mass conservation on time-dependent domains is often the starting point for various modelling efforts. A similar derivation of it is also given in Crampin et al. (1999). We will give specific choices for $\textbf{F}$ and *f* for our models later in §3.

#### Remark 1

We draw the attention of the reader to the fact that the above derivation of the conservation law (4) makes the implicit assumption that the velocity $\textbf{v}$ driving domain change is the same as the velocity with which mass is redistributed in the spatial domain due to that domain change. Any deviation from that, for instance caused by a domain change in which cells represented by density *u* are not fixed to the growing or shrinking substratum, must be accounted for in the expressions for flux or source density in that equation.

### Special Case: Spatially One-Dimensional Domains

We consider spatially one-dimensional models in this study; we briefly comment on the higher-dimensional case in §7. In this case, the domains $\Omega _t$ can be described by their left end point *l*(*t*) and their positive length *L*(*t*), *i.e.* $\Omega _t=(l(t),l(t)+L(t))$. This encompasses two typical scenarios of time-dependent spatial domains:

1. Type L: with left end fixed at zero, *i.e.* $l\equiv 0$ and $\Omega _t=(0,L(t))$ and
2. Type S: symmetric around $x=0$, *i.e.* $l(t)=-L(t)/2$ and $\Omega _t=(-L(t)/2,L(t)/2)$ .

The local rate of change in domain length becomes $\mathcal {J}_{x}\textbf{v}(t,x)=\partial _x v(t,x)$; note that now the velocity $\textbf{v}$ is a scalar field and so we drop the bold face in the notation). The domain length *L*(*t*) satisfies the following ODE

5 $$\begin{aligned} \dot{L}(t) = \int _0^{L(t)}\partial _x v(t,l(t)+x)\,\textsf{d}{x}\,. \end{aligned}$$

In the case of spatially homogeneous domain change we have that $\partial _x v(t,x)$ is independent of *x* and we denote $\partial _x v(t,x)=:r(t)$. Thus, from the above, $\dot{L}(t)= r(t)L(t)$ and also $v(t,x)=r(t)x+C(t)$ for some function *C*(*t*). To determine *C*(*t*) observe from (1) that, since $x(t;l(t_0))=l(t)$, we have $\dot{l}(t)=v(t,x(t;l(t_0)))$ and consequently $C(t) = \dot{l}(t)-r(t)l(t)$ and thus

6 $$\begin{aligned} v(t,x) = r(t)(x-l(t))+\dot{l}(t)=\frac{\dot{L}(t)}{L(t)}(x-l(t))+\dot{l}(t)\,. \end{aligned}$$

For both, Type L and Type S domains, and spatially homogeneous domain change this simplifies to

7 $$\begin{aligned} v(t,x) = r(t)x = \frac{\dot{L}(t)}{L(t)}x\,. \end{aligned}$$

Different regimes of spatial domain change can be realised. In Appendix A, we give expressions for *L*(*t*) and its derivative for linear, exponential and logistic-type change of domain length. In the case of spatially homogeneous domain change and with these expressions, the corresponding *r*(*t*) and *v*(*t*, *x*) can be computed using the formulas above. In addition, we discuss there that the same overall domain length *L*(*t*) can also be realised with a spatially heterogeneous domain change. This is particularly useful for investigating the impact of spatially homogeneous *vs.* heterogeneous domain change independent of the overall length change.

In the here considered case of spatially one-dimensional domains defined by known functions *l*(*t*) and *L*(*t*), we can easily transform the time-dependent domains to a fixed space-time domain using the change of variables

8 $$\begin{aligned} \hat{t}(t,x):=t \quad \text {and}\quad \hat{x}(t,x):=\frac{x-l(t)}{L(t)}\,, \end{aligned}$$

which maps $\Omega _I$ to $I\times \hat{\Omega }$ where $\hat{\Omega }:=(0,1)$ is time-independent. Defining the transformed density $\hat{u}:I\times \hat{\Omega }\rightarrow \mathbb {R}$ via

$$ u(t,x)=:\hat{u}(\hat{t}(t,x), \hat{x}(t,x))\quad \text {for } (t,x)\in \Omega _I\,, $$

we obtain that *t*- and *x*-derivatives of *u* (or of any other function defined on $\Omega _I$) transform as follows

9 $$\begin{aligned} \partial _t u(t,x) = \partial _{\hat{t}}\hat{u}(\hat{t},\hat{x}) -\frac{\dot{l}(\hat{t})+\dot{L}(\hat{t})\hat{x}}{L(\hat{t})}\partial _{\hat{x}}\hat{u}(\hat{t},\hat{x}) \quad \text {and}\quad \partial _x u(t,x) = \frac{1}{L(\hat{t})}\partial _{\hat{x}}\hat{u}(\hat{t},\hat{x})\,. \end{aligned}$$

Similarly to $\hat{u}$, we define velocity $\hat{v}$, flux function $\hat{F}$, and source density $\hat{f}$ with respect to the transformed coordinates. Then the conservation law (4) transforms to

10 $$\begin{aligned} \partial _{\hat{t}}\hat{u} = - \frac{1}{L}\partial _{\hat{x}}\left( \hat{F} + \hat{u} \left( \hat{v}-(\dot{l} +\dot{L} \hat{x})\right) \right) + \hat{f} -\frac{\dot{L}}{L}\hat{u}\,. \end{aligned}$$

The equation is again in conservative form but has a modified flux function and a modified source density.

In the case of spatially homogeneous domain change, we use (6) rewritten as $\hat{v}= \dot{L} \hat{x}+\dot{l}$ to simplify Eq. (10) and arrive at

11 $$\begin{aligned} \partial _{\hat{t}}\hat{u} = - \frac{1}{L}\partial _{\hat{x}}\left( \hat{F} \right) + \hat{f} -\frac{\dot{L}}{L}\hat{u}\,. \end{aligned}$$

The modification $-\dot{L} \hat{u} / L$ in the source density in Eqs. (10) and (11) corresponds,

- for a growing domain, *i.e.* $\dot{L}>0$, to a decrease in $\hat{u}$ (the conserved quantity is diluted by domain growth) and,
- for a shrinking domain, *i.e.* $\dot{L} <0$, to an increase in $\hat{u}$ (the conserved quantity increases concentration by domain shrinking).

#### Remark 2

In the special case of Type S domains, we can also transform the spatial domain using $\hat{x}(t,x):=x/L(t)$ such that $\Omega _I$ is mapped to the fixed domain $I\times \hat{\Omega }$ where $\hat{\Omega }:=(-1/2,1/2)$. Then (4) transforms to

12 $$\begin{aligned} \partial _{\hat{t}}\hat{u} = - \frac{1}{L}\partial _{\hat{x}}\left( \hat{F} + \hat{u} \left( \hat{v}-\dot{L} \hat{x}\right) \right) + \hat{f} -\frac{\dot{L}}{L}\hat{u} \end{aligned}$$

instead of (10). Under the additional assumption of homogeneous domain change, we again arrive at the formula as given in (11).

## A General One-Species Non-Local Model on a Time-Dependent Spatial Domain

As indicated earlier, we restrict in this study to the case of spatially one-dimensional domains. Here, in this section, we gather various terms which are frequently used to model local and non-local interactions of a cellular population; these generalise to multiple cell populations or also to the inclusion of an extracellular matrix (ECM) but this is of no concern here. We give these expressions, in their original form, with respect to the time-dependent spatial domain as well as, in their transformed form, with respect to the fixed spatial domain after applying transformation (8); the transformed form is also valid in case of the coordinate transform discussed in Remark 2. In the end of this section we then combine these terms to state our general one-species non-local model on a time-dependent spatial domain in its original and transformed form.

***Cell-random motility***

Cell-random motility is often represented by Fickian diffusion in models and the corresponding flux term reads, in its original and its transformed form using (9),

13 $$\begin{aligned} F_{\text {(d)}}(t,x) := -D\partial _x u(t,x) \quad \text {and}\quad \hat{F}_{\text {(d)}}(\hat{t}, \hat{x}) := -\frac{D}{L(t)}\partial _{\hat{x}} \hat{u}(\hat{t},\hat{x})\,, \end{aligned}$$

where $D>0$ is the *diffusion coefficient*, which we here assume independent of *t* and *x*.

Different forms of nonlinear diffusion have also been considered as models of cell random motility and we in particular mention models of cross-diffusion which become important when considering multiple interacting cell types or in the case of interactions with the ECM, see, for instance, Murakawa and Togashi (2015); Madzvamuse et al. (2017); Carrillo et al. (2019).

***Cell proliferation***

Cell proliferation is frequently modelled by a logistic growth term and the corresponding source density reads, in its original and its transformed form,

14 $$\begin{aligned} f_{\text {(p)}}(t,x) := \rho u(t,x) \left( 1-\frac{u(t,x)}{U}\right) \quad \text {and}\quad \hat{f}_{\text {(p)}}(\hat{t},\hat{x}) := \rho \hat{u}(\hat{t},\hat{x}) \left( 1-\frac{\hat{u}(\hat{t},\hat{x})}{U}\right) \,, \end{aligned}$$

where $\rho >0$ is the *proliferation rate* and $U>0$ is the *carrying capacity*.

The constant *U* typically reflects the fact that growth becomes increasingly restricted when *u* approaches *U* from below due to nutrient depletion. However note that also competition for space may restrict cell proliferation, in particular in the case of multiple interacting cell types or in the case of interactions with the ECM; in that case different limiting factors may arise in the cell proliferation term, see for example (Domschke et al. 2014).

***Non-local attracting and repelling interactions***

For modelling non-local attracting or repelling interactions between the cells we use the non-local expression as discussed in Painter et al. (2015) which is based on the expression originally proposed by Armstrong et al. (2006). The resulting flux is a product of four factors and reads

15 $$\begin{aligned} F_{\text {(n)}}(t,x) := u(t,x) \cdot p(u(t,x)) \cdot \omega \cdot \mathcal {A}\{u(t,\cdot )\}(x)\,. \end{aligned}$$

The first factor is the density *u* itself and the product of the following three factors gives the velocity with which the quantity represented by *u* is moved due to non-local attracting or repelling interactions. The second factor, *p*(*u*(*t*, *x*)), is the so-called *packing* or *volume-filling function*, a non-increasing function of *u* which models the effect of the decreased potential of cells to move in higher density environments. Following Painter and Hillen (2002), we take the packing function in the following form

16 $$\begin{aligned} p(u):=1-\frac{u}{P}\,, \end{aligned}$$

where $P>0$ is the *limiting packing density* or we take it as $p(u)\equiv 1$, *i.e.* we ignore volume-filling effects. In (16) we have $p(u)<0$ for $u>P$ and a change of sign results in (15) implying a change from attractive to repelling behaviour or *vice versa*. In such a situation the following modified form of (16) might be more appropriate

17 $$\begin{aligned} p(u):=\max \left\{ 0,1-\frac{u}{P}\right\} \end{aligned}$$

and in our simulations we use this form instead of (16). The third factor, $\omega >0$, is a parameter which itself depends, *e.g.*, on the viscosity of the surrounding medium and the cell diameter as discussed in Armstrong et al. (2006). The fourth and final factor, $\mathcal {A}\{u(t,\cdot )\}(x)$, is the non-local interaction term evaluated at *x* and is defined as

18 $$\begin{aligned} \mathcal {A}\{u(t,\cdot )\}(x):=\int _{-\xi }^\xi \frac{r}{|r|}\Omega (|r|; \xi ,\mu )g(u(t,x+r))\,\textsf{d}{r}\,. \end{aligned}$$

Here, $\xi >0$ is the *sensing radius* determining the *sensing region* $x+[-\xi ,\xi ]$ around *x* over which cells at *x* can sense their environment and interact in an attracting or repelling fashion. Next, *r*/|*r*| is the unit vector pointing from *x* to the point of interaction $x+r$ within the sensing region and encodes the directional information. The function $g(u(t,x+r))$ represents the effect of the sensing at $x+r$ on the cells at *x*. We here take it simply as $g(u):=u$, *i.e.* a higher density of *u* at the point of interaction $x+r$ leads to a stronger effect on the cells at *x*. Note that also in function *g* we can model volume-filling effects on the non-local interactions, see Gerisch and Chaplain (2008). Finally, $\Omega (|r|; \xi ,\mu )$ is the *interaction function*^2^ which characterises the strength of the resulting effect on cells at *x* by interaction with cells at $x+r$—within the sensing region—as a function of their distance $|r|\le \xi$. It takes the form

19 $$\begin{aligned} \Omega (|r|; \xi ,\mu ):=\mu \frac{\tilde{\Omega }(|r|/\xi )}{\xi }\,,\text { where } \int _0^\xi \tilde{\Omega }(r/\xi )\,\textsf{d}{r}=\xi \,, \end{aligned}$$

and $\tilde{\Omega }:[0,1]\rightarrow \mathbb {R}$ is the *normalised interaction function*. We only consider normalised interaction functions with non-negative values here such that the *interaction strength parameter* $\mu$ determines, both, the strength of the interaction and whether the interaction is attracting ($\mu > 0$) or repelling ($\mu <0$). We consider the most simple form of normalised interaction function in this work, namely a constant one on $[0,\xi ]$, taking the form

20 $$\begin{aligned} \tilde{\Omega }(|r|/\xi ):={\left\{ \begin{array}{ll} 1 & :\quad |r|/\xi \le 1\\ 0 & :\quad |r|/\xi > 1 \end{array}\right. }\,. \end{aligned}$$

### Remark 3

Note that the normalisation condition in (19), as given in Painter et al. (2015), differs slightly from that proposed in Gerisch and Chaplain (2008). That needs to be taken into account when comparing models and results; it can be compensated by choosing the interaction strength parameter in Gerisch and Chaplain (2008) as $2\xi \mu$ where $\xi$ and $\mu$ are as in Painter et al. (2015).

We finally note that we consider models with periodic BCs in this study, see also below, such that the non-local term (18) is also well-defined near any spatial domain boundary.

Changing coordinates from the time-dependent to a fixed spatial domain, the flux expression (15) together with the non-local term expression (18) change as follows

21a $$\begin{aligned} \hat{F}_{\text {(n)}}(\hat{t},\hat{x})&:= \hat{u}(\hat{t},\hat{x}) \cdot p(\hat{u}(\hat{t},\hat{x})) \cdot \omega \cdot \,\,\hat{\mathcal {A}}\{\hat{u}(\hat{t},\cdot )\}(\hat{x})\,,\end{aligned}$$

21b $$\begin{aligned} \hat{\mathcal {A}}\{\hat{u}(\hat{t},\cdot )\}(\hat{x})&:=L(\hat{t})\int _{-\xi /L(t)}^{\xi /L(t)}\frac{\hat{r}}{|\hat{r}|}\Omega (|\hat{r}|L(t); \xi ,\mu )g(\hat{u}(\hat{t},\hat{x}+\hat{r}))\,\textsf{d}{\hat{r}}\,, \nonumber \\&=\int _{-\hat{\xi }(\hat{t})}^{\hat{\xi }(\hat{t})}\frac{\hat{r}}{|\hat{r}|}\Omega (|\hat{r}|; \hat{\xi }(\hat{t}),\mu )g(\hat{u}(\hat{t},\hat{x}+\hat{r}))\,\textsf{d}{\hat{r}}\,. \end{aligned}$$

In the above, we have used the notation $\hat{r}:=r/L(t)$ and $\hat{\xi }(\hat{t}):=\xi /L(t)$, *i.e.* these quantities transform in the same way as *x*. Furthermore, for the last equality we have used (19) to observe $L(\hat{t})\Omega (|\hat{r}|L(t); \xi ,\mu ) = \Omega (|\hat{r}|; \hat{\xi }(\hat{t}),\mu )$.

***A one-species non-local model on a time-dependent spatial domain*** We now use in (4) the above expressions for the fluxes, representing cell random motility and non-local interactions, as well as the source density, representing cell proliferation, to state our general one-species non-local model on a spatially one-dimensional time-dependent spatial domain together with an IC prescribed through an initial function $u_0(x)$ and periodic BCs as

22 $$\begin{aligned} \begin{aligned}&\partial _t u =-\partial _x\left( uv-D\partial _x u+up(u)\omega \mathcal {A}\{u(t,\cdot )\} \right) +f_{\text {(p)}}\quad \text {for }x\in \Omega _t\,, t\in (t_0,T]\,,\\&\text {subject to IC } u(t_0,x) = u_0(x)\quad \text {for }x\in \Omega _{t_0} \text { and periodic BCs}\,. \end{aligned} \end{aligned}$$

Transforming to a fixed spatial domain, and under the assumption of a spatially homogeneous domain change, we arrive using (11) at

23 $$\begin{aligned} \begin{aligned}&\partial _{\hat{t}} \hat{u} =-\frac{1}{L}\partial _{\hat{x}}\left( \!\!-\frac{D}{L}\partial _{\hat{x}} \hat{u}+\hat{u}p(\hat{u})\omega \,\,\,\hat{\mathcal {A}}\{\hat{u}(\hat{t},\cdot )\}\!\!\right) +\hat{f}_{\text {(p)}}-\frac{\dot{L}}{L} \hat{u}\text { for }(\hat{t},\hat{x})\in (t_0,T]\times \hat{\Omega }\,,\\&\text {subject to IC } \hat{u}(t_0,\hat{x}) = \hat{u}_0(\hat{x})\quad \text {for }\hat{x}\in \hat{\Omega }\text { and periodic BCs}\,. \end{aligned} \end{aligned}$$

Later we will also have a look at the cell mass *m*(*t*) in all of $\Omega _t$ at time *t*, given by

24 $$\begin{aligned} m(t):=\int _{\Omega _t} u(t,x)\,\textsf{d}{x} = L(\hat{t}) \int _{\hat{\Omega }} \hat{u}(\hat{t},\hat{x})\,\textsf{d}{\hat{x}}\,. \end{aligned}$$

## Numerical Approach

The numerical approach taken to simulate a non-local model of the form (22) on a time-dependent spatial domain, after it has been transformed to a fixed spatial domain yielding (23), essentially follows that described in Gerisch and Chaplain (2008) and with more detail regarding the non-local term approximation and evaluation in Gerisch (2010). We follow the Method of Lines (MOL) and use an appropriate Finite Volume scheme for the discretization in space of the transformed model equation on a spatially uniform mesh of the fixed spatial domain. The time integration of the resulting MOL-ODE, with automatic time-step size control, is subsequently done using the highly efficient, 4th-order, linearly-implicit Runge-Kutta scheme ROWMAP, see Weiner et al. (1997). In all simulations presented in this work, we use this solver with its absolute and relative tolerance parameter set to the rather strict value of $10^{-7}$. The computational bottleneck of this approach is the evaluation of the non-local term (21b) on all grid cell interfaces of the spatial mesh at a given time point $t\in I$. This evaluation is required many times over the course of a simulation, in fact it is required at any time point where the time integration scheme needs the evaluation of the right-hand side of the MOL-ODE. We discuss this particular issue in the remainder of this section.

In the case of a *fixed* *spatial domain*, covered by a uniform spatial mesh, and periodic BCs for the model equation this bottleneck can be circumvented by observing that the simultaneous evaluation of the non-local term on all grid cell interfaces at a time point $t\in I$ amounts to a matrix-vector product with a circulant matrix of dimension, say, $N\times N$. This matrix is not sparse, not even for a sensing radius $\xi$ which is small compared to the spatial domain size, and thus the matrix-vector product naively requires $\mathcal {O}({N^2})$ operations. Thanks to the circulant structure of the matrix, this can be reduced to $\mathcal {O}({N\log N})$ operations when using the Fast Fourier Transform (FFT) algorithm. Two other important features of this circulant matrix are

1. that the matrix is independent of time *t* and thus can be pre-computed to any desired accuracy before the actual time integration starts, and
2. that the matrix is fully defined by its first column, so that only at most *N* entries need to be computed and stored.

This essentially implies that the evaluation (on all grid cell interfaces) of the non-local flux expression (21a) is only marginally more expensive than the evaluation (on all grid cell interfaces) of the diffusive flux expression (13). This removes the computational bottleneck and renders the simulations highly efficient.

In the case of a *time-dependent domain with spatially homogeneous domain change*, the focus of this work, not all of the above nice features can be retained. After the transformation to a fixed spatial domain $\hat{\Omega }$, we still have periodic BCs and we also use a uniform spatial mesh on $\hat{\Omega }$. Thus, the simultaneous evaluation of the non-local term (21b) on all grid cell interfaces still amounts to a matrix-vector product with a circulant matrix of dimension, say, $N\times N$. The difference, however, is that the integration limits of the non-local term (21b) now depend on the time point $t=\hat{t}$. This makes the pre-computation of the required at most *N* entries of this matrix impossible and rather requires to compute this matrix entries anew whenever the non-local term is to be evaluated. Luckily, this can be accomplished, *e.g.* for the normalised interaction function (20), at constant cost per entry, *i.e.* at total cost $\mathcal {O}({N})$ for the full first column of the circulant matrix. This cost is less than the $\mathcal {O}({N\log N})$ cost of the subsequent evaluation of the matrix-vector product for which we can still use the efficient FFT algorithm. Taken together, we can expect a slight increase in computation time for a simulation of the non-local model on a time-dependent domain with spatially homogeneous domain change—compared to the computation time for a simulation on a fixed spatial domain. This is also what we observe in practice.

### Remark 4

The situation becomes significantly different, however, for the case (here not further considered) of a time-dependent domain with *spatially heterogeneous domain change*, even under the assumption of periodic BCs and of a uniform spatial mesh on $\hat{\Omega }$. Here not only the matrix becomes time-dependent but it also looses its circulant structure due to the heterogeneity of the domain change. As a consequence $\mathcal {O}({N^2})$ matrix entries must be computed for each evaluation of the non-local term on all grid cell interfaces (at each time point required). Furthermore, the evaluation of the matrix-vector product also costs $\mathcal {O}({N^2})$ operations since the FFT cannot be used due to the lack of the circulant matrix property. In practical terms this means that a simulation of a model on a spatially one-dimensional time-dependent domain with spatially heterogeneous domain change costs about as much as a simulation of a similar model but on a two-dimensional fixed spatial domain.

## An Aggregating Cellular Population

### Model Formulation

Here we consider model (22), or its transformed fixed spatial domain version (23), for a self-attracting ($\mu >0$) and non-proliferating ($f_{\text {(p)}}\equiv 0$) cell population on an exponentially growing ($\alpha >0$) Type L domain. In the non-local term we use the packing function (17), the normalised interaction function (20), and as discussed earlier $g(u):=u$. We fix the following parameters

25 $$\begin{aligned} t_0=0\,,\quad T=10\,,\quad L_0=2\,,\quad D=10^{-3}\,,\quad \omega =1\,,\quad P=0.8\,,\quad \xi =0.4\,. \end{aligned}$$

Note that we do not specify units here as the focus of this work is on demonstrating generic behaviour of the models rather than reproducing or predicting a particular biological scenario. The remaining parameters $\alpha$ (rate of domain growth) and $\mu$ (interaction strength) are selected later and reported in the numerical experiments section §5.2 below. The initial condition is chosen as $u_0(x):=P/3+10^{-3}\textsf {U}_{(-1,1)}(x)$, *i.e.* as a third of the limiting packing density plus a uniformly distributed spatial perturbation of magnitude $10^{-3}$. A model of this form, but with packing function equal to one and without a normalisation of the interaction function, has been considered in Armstrong et al. (2006) for the case of a fixed spatial domain, thus providing a point of reference for our model on a growing domain.

### Simulation Results

For the model specified in §5.1 we focus on simulations which illustrate aggregation of cells starting from a perturbed homogeneous steady state (pattern formation) and discuss the impact of the growing domain on the model solution in comparison to solutions from the model stated on a fixed bounded domain. For this, a Fourier-type stability analysis can give insight into which Fourier modes in an initial perturbation are amplified and give rise to pattern formation.

An analysis of this kind for our model, with minor modifications, on a *fixed spatial domain equal to the full real line* is given in (Armstrong et al., 2006, Section 3.2). Notably it is shown there that patterns emerge, *i.e.* that cells aggregate in clusters, as soon as the interaction strength parameter $\mu$ has a sufficiently large value.

We present this Fourier-type analysis for our model, but on a *fixed spatial domain equal to* (0, *L*) *with periodic boundary conditions* and give details in Appendix B. Again we can state that for sufficiently large $\mu \ge \mu _\text {min}$ cell aggregation takes place. Furthermore, the domain length *L* is now an additional parameter influencing the emergence of patterns. We observe, for the model parameters selected in this section, that the value of $\mu _\text {min}$ does not depend much on *L*, see Table 2 in Appendix B, while with increasing *L* the dominant Fourier mode has increasing wave number, *i.e.* solutions with more clusters can emerge, see Table 3 in Appendix B.

A Fourier-type stability analysis for our model on a *time-dependent spatial domain with periodic boundary conditions* is beyond the scope of this contribution and deferred to future work. Nevertheless, from the conclusions drawn in Appendix B, we can already infer some insight into this case as well.

#### Domain growth slows down cellular aggregation

**Fig. 1:**
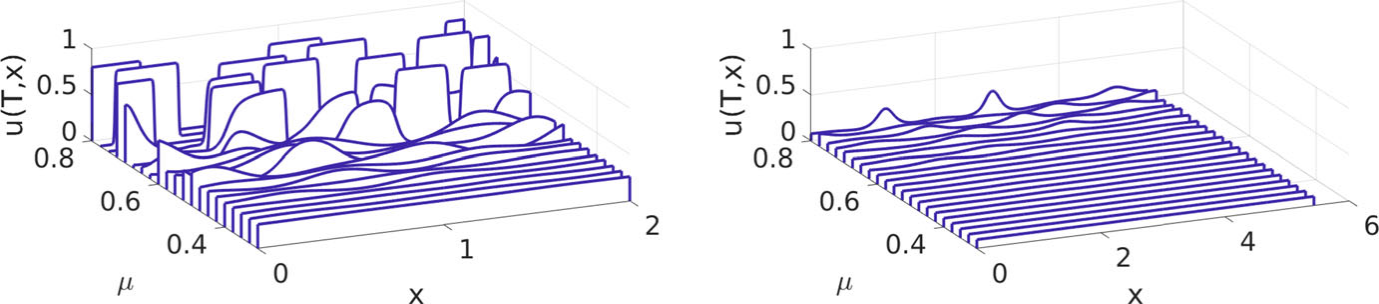
Numerical solution of the cellular aggregation model without domain growth ($\alpha =0$, left plot) and with exponential domain growth ($\alpha =0.1$, right plot). In each plot we show the numerical approximation of *u*(*t*, *x*) at final time $t=T$ for a selection of values of the interaction strength parameter $\mu$

We consider the model specified in §5.1 and compare simulated solutions at final time $T=10$ for a selection of values $\mu \in [0.3, 0.8]$. We do this for the model on a fixed spatial domain (simply chose $\alpha =0$), shown in the left plot of Fig. 1, and on an exponentially growing domain with parameter $\alpha =0.1$, shown in the right plot of Fig. 1.

The growth of the domain in the second case leads to a spatial domain length of $L(T)\approx 5.4$ at final time $T=10$, *i.e.* an almost tripling of the initial domain length $L_0=2$.

Before discussing the observations from these simulations, we provide a bit more insight into the numerics here. Firstly, these and all other simulations in this subsection are performed on a fixed uniform spatial grid covering the transformed fixed spatial domain $\hat{\Omega }=(0,1)$ with $N=1500$ grid cells. This means that the effective grid cell length in the untransformed domain is initially (at $t=0$) about $1.3\times 10^{-3}$ and increases on the growing domain to about $3.6\times 10^{-3}$ at final time $T=10$. So we have a sufficiently fine grid over the whole time interval and in particular also sufficiently many grid cells within the sensing region of the non-local term of size $2\xi =0.8$. Secondly, the simulations of the model equation for each value of $\mu$ take about 2 to 3 seconds on a standard laptop and are thus highly acceptable.

From the stability analysis in Appendix B, see Table 2, we obtain that for a fixed spatial domain of length $L=2$ we will observe pattern formation if $\mu \ge \mu _\text {min}\approx 0.011$. However, for such small values, aggregation is still very slow and not yet visible at $T=10$. In the left plot of Fig. 1 we observe that on the fixed spatial domain the cells aggregate visibly over this time period if $\mu >0.4$ and they do so strongly if $\mu >0.6$. We also observe for $\mu >0.6$ that the cell aggregates at $T=10$ reach a limiting maximum density at roughly the value of parameter $P=0.8$ of the packing function and merging of cell aggregates and plateau formation have taken place. The latter is in contrast to the simulation results presented in (Armstrong et al., 2006, Fig. 4), where the model has no packing function and thus the aggregation of the cells is not limited and pushes the cell density to larger values.

The right plot of Fig. 1 shows the simulation results for an exponentially growing domain with domain length increasing from initially $L(0)=2$ to $L(10)\approx 5.4$. Over this range of domain lengths, the model on a fixed spatial domain, has a threshold for aggregation $\mu _\text {min}$ which is only minimally changing with *L*, see Table 2. The onset of aggregation is visible in the plot only for $\mu$ larger than $\approx 0.6$ and is at the same final time $T=10$ in magnitude considerably smaller compared to the case of the fixed spatial domain shown in the left plot of Fig. 1. This is attributed to the diluting effect of domain growth on the cell density which works against aggregation. From this we conclude: *domain growth slows down cellular aggregation*.

#### Domain growth allows for the emergence of cellular clusters

**Fig. 2:**
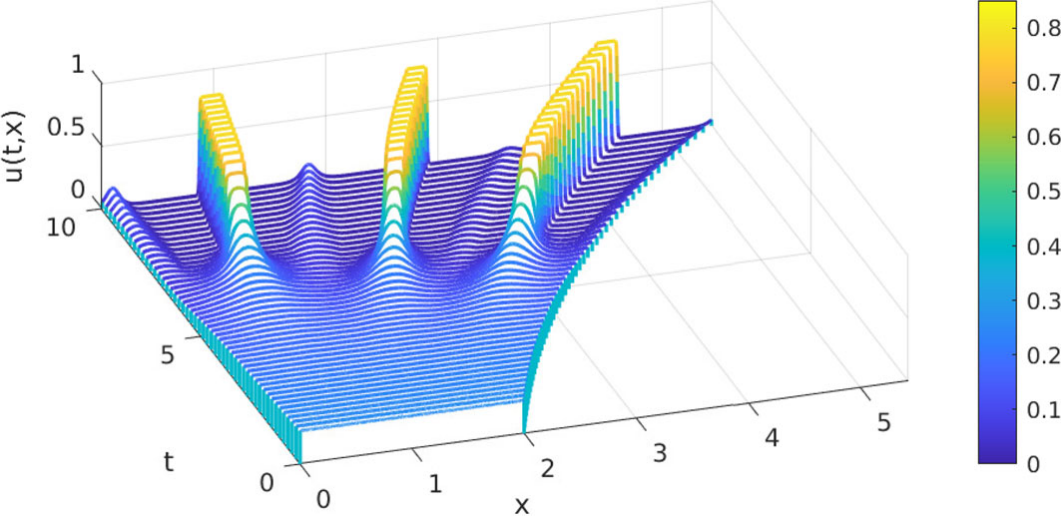
Numerical approximation of the solution *u*(*t*, *x*) of the cellular aggregation model with exponential domain growth ($\alpha =0.1$) and interaction strength parameter $\mu =1.2$

In Fig. 2 we show the spatio-temporal evolution of the numerical solution of the model specified in §5.1 on the same exponentially growing domain ($\alpha =0.1$) but with a larger interaction strength parameter $\mu =1.2$ as used in the simulations discussed in §5.2.1.

Between $t=3$ and 4 we see the onset of cellular aggregation which quickly develops into the formation of three distinct cellular clusters. These clusters approach the limiting packing density $P=0.8$ and plateau formation sets in with their width mainly dictated by this *P* and the cell mass in each cluster. As the spatial domain grows with time these clusters also increase their mutual distance. Domain growth by itself also leads to an increase of the plateau width and, by the diluting effect of domain growth, a decrease in their height (cell density). However, these effects are counteracted instantaneously by renewed aggregation within each cluster such that we observe that clusters maintain their width and height while changing position in space.

The initial emergence of three clusters is supported by the Fourier-type stability analysis as shown in Appendix B, see in particular Table 3. There the dominant wave numbers are given for the model as considered here but for various fixed domain lengths. For the domain length corresponding to $t=3$, where we see the onset of aggregation in Fig. 2, the dominant wave number is $k=3$ and we should expect the formation of three cell clusters as is indeed the case.

At around $t=7$ we see the emergence of new smaller cellular aggregates in between neighbouring clusters. This can be understood from two different mechanisms in the model and both are triggered by the increasing domain length.

Firstly, by this time the distance between neighbouring clusters becomes larger than two times the sensing radius $\xi =0.4$. This implies that there is a spatial region in the centre between two such clusters which no longer impacts the non-local term evaluated in these clusters. Thus, any remaining cells (recall that we have no cell proliferation in this model) around this spatial region can now aggregate into a new cluster. Secondly, that these small new clusters persist and can grow, is also supported by the Fourier-type stability analysis in Appendix B. From Table 3 we read that for a domain length corresponding to $t=8$ the dominant wave number is $k=6$ and thus solutions with six cell clusters are supported. These are seen in Fig. 2. While both mechanisms have their role in the emergence of the new clusters, we anticipate that the first mechanism is the dominant one since at around $t=7$ the solution is already far away from a homogeneous steady state which is the starting point for the Fourier-type analysis.

## Neural Crest Cell Migration

### Model Formulation

The model of NC cell migration is taken as in Painter et al. (2015) for a fixed spatial domain, again in order to provide the opportunity to later compare between growing and fixed domain simulations. This model also fits into the general model (22), or its transformed fixed spatial domain version (23). In the model, the process of contact inhibition of locomotion of NC cells is modelled via a non-local repelling term ($\mu <0$) for the NC cell density *u*. The cells are proliferating and so we set $f_{\text {(p)}}$ as given in (14). In the non-local term we use the normalised interaction function (20) and as discussed earlier $g(u):=u$. In Painter et al. (2015) the packing function *p*(*u*) as given in (16) is used. However, we will use the modified version (17) and also consider the choice $p\equiv 1$, essentially setting $P=+\infty$ in (17), and will compare both cases. The reason behind the latter choice is that in a repelling cell population, as considered here, it is not *a priori* clear why the cell flux due to this repelling interaction should be limited, or even vanish, in densely populated areas—one could rather expect a speedy dispersal of cells in such regions. We consider the model on an exponentially growing ($\alpha >0$) Type S domain with an initial cluster of NC cells around $x=0$. This is captured by the following initial condition

26 $$\begin{aligned} u(t_0,x) = {\left\{ \begin{array}{ll} U_0 & \text { if } |x|\le x_0\\ 0 & \text { otherwise} \end{array}\right. }\,. \end{aligned}$$

We take the following parameters from Painter et al. (2015)

27 $$\begin{aligned} \begin{gathered} t_0=0\,,\quad T=240\,,\quad L_0=11\,,\quad D=3.6\times 10^{-4}\,,\quad \omega =1\,,\quad P=10^4\,, \\ \xi =0.01\,,\quad \mu =-10^{-3}\,,\quad \rho =0.07\,,\quad U=10^3\,,\quad U_0=U\,,\quad x_0=0.05\,. \end{gathered} \end{aligned}$$

Note that the values for the sensing radius $\xi$ and the interaction strength parameter $\mu$ correspond to the parameter choice in subplot (a3) of Fig. 9 in Painter et al. (2015) and they are taken from the ranges [0.01, 0.1] and $[-10^{-3},0]$, respectively, as considered in that paper. The dimensionality of these parameters is such that lengths are in millimetres and time is in hours, such that we observe the process over 10 days. We note, as stated and explained in Painter et al. (2015), that while these parameter “values are not rooted in solid data, they are by no means unreasonable”. The specific values of the parameter $\alpha$ (rate of domain growth) as well as different values of the parameter *P* of the packing function are selected in §6.2 when presenting particular simulations.

### Simulation Results

We consider the model as detailed in §6.1 which has already been considered—on a fixed spatial domain—in Painter et al. (2015) with the same set of parameters. For simulations on a fixed spatial domain we set $\alpha =0$ while for growing domain simulations we use $\alpha =0.007$. In the latter case, the growing domain has, at final time $T=240$ a domain size of $11\exp (\alpha T) \approx 59$, *i.e.* it grows by a factor of about 5.4 over this 10-day period.

**Fig. 3:**
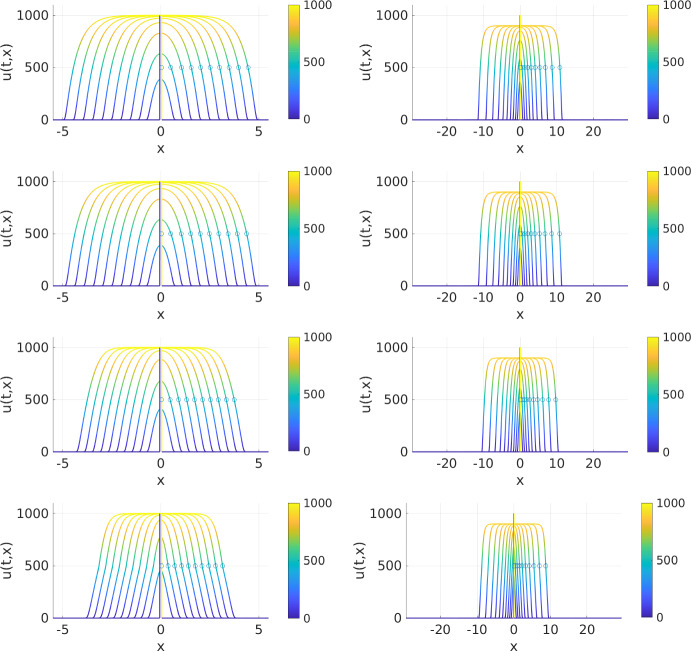
Numerical approximation of the solution *u*(*t*, *x*) of the NC cell migration model without domain growth ($\alpha =0$, left column plots) and with exponential domain growth ($\alpha =0.007$, right column plots) and for packing function $p\equiv 1$ (1st row plots) and *p*(*u*) as in (17) with $P=10^4$ (2nd row plots), $P=10^3$ (3rd row plots), and $P=5\cdot 10^2$ (4th row plots). The solution curves shown in each plot are 24 hours apart and are moving to the left and right from around $x=0$; on the right-moving fronts, the numerically identified front position is marked with a circle

The two plots in the second row of Fig. 3 show simulation results of the model with parameter values as in (27) on the fixed spatial domain (left column plot) and on the exponentially growing spatial domain (right column plot); the other rows in that figure are obtained with different parameter values *P* and are discussed in §6.2.1. In both plots, the solution spreads out from its initial peak around $x=0$ to the left and right in a travelling wave-like manner. We plot the solution curves at time points one day apart, so at $t=0, 24, 48,\dots$. Note that in the right plot the *x*-scale is much larger due to domain growth. In each solution curve we mark with a circle the position of the invading front, defined as, *cf.* Painter et al. (2015), the *x*-value where the right-moving part of *u* takes on the value of *U*/2. There are two observations to be made from these simulations and plots:

- On the fixed spatial domain the solution reaches up to the carrying capacity $U=10^3$ of the proliferation term while on the growing spatial domain the maximum is well below. This is again caused by the diluting effect of domain growth on the cell density.
- On the growing spatial domain, NC cells occupy an about twice as large spatial region compared to the fixed domain case. Below in §6.2.2 we will identify how much of that larger occupied region is directly due to domain growth and how much due to migratory movement of NC cells (which may increase or reduce compared to the fixed domain case as an indirect result of domain growth). What we see here already, from the marks of the invading front, is that on the fixed spatial domain the invading front appears to move with a constant velocity (markers are equispaced), while on the growing domain we have that overall the velocity is increasing with time.

#### The effect of the packing function on tissue invasion

In the description of the NC cell migration model in §6.1 we proposed that, due to the repelling nature of the non-local term in that model, the use of the packing functions *p*(*u*) as in (16) or (17), which limit cell movement in highly packed spatial regions, might appear counter-intuitive or not even be justified. We will have a closer look at this now. Note that the discussion in this section is relevant for models with a repelling non-local term in both, fixed and growing domain simulations, and we thus do not focus too much on the differences due to the domain type.

In the simulations of the NC cell migration model as shown in the second row of Fig. 3, the packing function (17), *i.e.* $p(u)=\max \{0,1-u/P\}$ is used with parameter $P=10^4$. However, as can be seen from the simulation results, the maximum cell density in the model is essentially controlled by the proliferation term which limits the density to about the carrying capacity $U=10^3$, which is not even reached in the case with domain growth. The packing function thus takes values in [0.9, 1] and consequently the cell flux due to the non-local repelling term is reduced by at most 10% by the packing function and that only in the most dense areas. As a result, removing the packing function by letting $p(u)\equiv 1$ has negligible impact on the simulation outcome (results shown in first row of Fig. 3).

The situation changes when the difference in magnitude between *P* and *U* vanishes, we consider $P=10^3$ with results shown in the third row of Fig. 3, or is reversed, we consider $P=5\cdot 10^2$ with results shown in the fourth row of Fig. 3; in both cases we keep $U=10^3$ at its previous value. Now the impact of the limiting behaviour of the packing function becomes stronger and more visible and leads to a reduced invasion depth on fixed as well as on growing domains. We note that in the case of $P=5\cdot 10^2$ the cell density grows well beyond that limiting value due to the strength of the proliferation term.

We summarise by stating that, for fixed domain as well as for growing domain simulations, a movement-limiting packing function such as (17) can have an impact on the invasion of repelling cell populations; it is up to the modeller to decide whether this is biologically justified or rather the packing function $p\equiv 1$, *i.e.* no limiting on movement, should be used. For the NC cell migration model considered here and with parameters for $P=10^4$ and $U=10^3$ as taken from and used in Painter et al. (2015), the choice between these two packing functions has only negligible impact on the simulation results.

#### The effect of domain growth on tissue invasion

Since NC cells are moving with the growing tissue in our model, there is clearly a direct impact of domain growth on the size of the invaded tissue region. However, tissue growth may also have an indirect effect on the invasion depth by impacting the balance between the other terms in the model. We look into aspects of these indirect effects by (i) comparing the invading front positions between the fixed and growing domain situation over time, where we discount for domain growth in the latter case, and (ii) comparing the total cell mass in the domain between the fixed and growing domain situation over time.

***Domain growth leads to reduced migratory movement of NC cells***

The invading front positions as shown by the circles in Fig. 3, right column plots, include the direct effect of the exponentially growing domain. If an invading front is at time *t* in position *x* then, by tracing the domain growth trajectory backwards, this position was at position $x_0(t,x):=x\exp (-\alpha (t-t_0))$ at initial time $t_0$. We call this $x_0(t,x)$ the *invading front position discounted for domain growth at time* *t*.

**Fig. 4:**
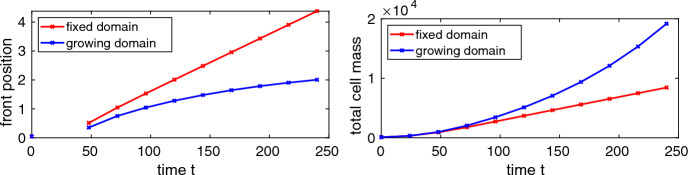
Numerically computed invading front positions in the fixed domain case and invading front positions discounted for domain growth in the growing domain case over time (left plot) and total NC cell mass over time (right plot) of the NC cell migration model with parameters as in the second row of Fig. 3. Note that we do not have front positions for $t=24$ as the cell density is below the threshold *U*/2 at this *t* and thus no front is detected.

We show in the left plot of Fig. 4 the invading front positions in the fixed domain case and the invading front positions discounted for domain growth in the growing domain case over time for the simulations with the same parameters as used in the second row of Fig. 3. We observe that the discounted front positions in the growing domain case are, over time, consistently and substantially smaller than the front positions in the fixed domain case. For example, at final simulation time the invading front position in the fixed domain simulation is a bit larger than four while the invading front position discounted for domain growth in the simulation with domain growth is about two, *i.e.* about 50% reduced. From this observation we imply that domain growth appears to impede the inherent invasive capabilities of the cells. This observation holds true not only for the particular choice of interaction strength parameter $\mu$ and proliferation rate $\rho$ used for this simulation but also if these are changed independently over one order of magnitude up or down (results not shown). A possible explanation of this observation is that in the growing domain case we have a density diluting effect due to that growth and this results in a weaker non-local interaction, *i.e.* less cellular repulsion and thus less invasion. This effect is to some extend counteracted by increased cell proliferation (dilution leads to cell densities further below the carrying capacity *U*) but the negative impact on cellular repulsion appears to dominate.

***Domain growth leads to increased NC cell proliferation***

The NC cell mass *m*(*t*) at time *t* in the full spatial domain is computed according to (24) which is approximated using the numerical simulation results. We show this mass as a function of time in the right plot of Fig. 4 for the fixed and growing domain cases. Here we observe that the total cell mass growths faster in the growing domain scenario. This observation is in line with the observations above and can again be attributed to the cell density diluting effect of spatial domain growth, driving the cell density further below the carrying capacity, and consequently leading to increased cell proliferation.

We summarise the effects of domain growth on tissue invasion by NC cells. It leads to an increased NC cells mass in the spatial domain and also to a larger invaded domain region compared to a fixed domain scenario. However, looking at the invaded region (front position) when discounting for the direct effect of domain growth, we observe that NC cells show reduced migratory movement in the growing domain compared to the fixed domain scenario.

## Conclusion

In this contribution we have presented a framework to model and simulate non-local models of collective cell migration on time-dependent spatial domains. We have restricted ourselves to spatially one-dimensional problems and spatially homogeneous domain change as the principle numerical challenges become clear in this simple setting already. We conclude in particular that such models can be simulated efficiently by the proposed numerical technique. Furthermore the considerations in terms of modelling and simulation documented here can form the basis for more advanced models which require a dedicated numerical treatment. The impact of time-dependent spatial domains, in comparison with fixed spatial domains, on the behaviour of model solutions has been investigated for a simple cellular aggregation model and a model of NC cell migration. Simulations show that the strongest impact on model solutions appears to be caused by the diluting effect of domain growth on the cellular density. This leads to secondary effects including a slowed down emergence of spatial patterns, a reduced migratory movement of NC cells, and an increased rate of cell proliferation. We have also seen by a Fourier-type stability analysis of the cellular aggregation model on fixed spatial domains of variable length that domain length impacts in particular the selection of the dominant wave number. While a Fourier-type stability analysis in the time-dependent domain case is beyond the scope of this work, and is deferred to the future, from the limited analysis presented we can draw conclusions about solution behaviour which are, nevertheless, consistent with the simulations on time-dependent spatial domain.

We have discussed the additional computational complexity when considering spatially heterogeneous as opposed to homogeneous domain growth in Remark 4 already. To overcome this complexity barrier, we propose to investigate a project-evaluate-interpolate approach: project a given numerical solution on a non-uniform spatial grid to a uniform grid, efficiently evaluate the non-local term on that uniform grid, and finally interpolate the non-local term to non-uniform grid positions as needed.

Furthermore, when going from a spatially one-dimensional setting to a higher-dimensional one, then a transformation to a time-independent reference domain might not be readily available. However, for the case of a rectangle or cuboid under spatially homogeneous growth this is possible and simulations, using a numerical approach employing the same ideas as in the one-dimensional setting here, are computationally feasible.

In this work we have also restricted to the case that the function $\textbf{v}$ driving domain change is known *a priori*. In many models this function $\textbf{v}$ will be a solution of another PDE itself and thus frequently lead to spatially heterogeneous domain change with some sub-regions growing and others shrinking. In this case the PDE for $\textbf{v}$ can be used to transform the full PDE system from the current configuration to the reference configuration, typically the spatial domain at initial time. This transformation, although not restricted to the spatially one-dimensional case, comes with its own challenges, in particular if the spatial domain is strongly deformed and might require an incremental approach with a sequence of reference domains at increasing time points.

## Figures and Tables

**Fig. 5:**
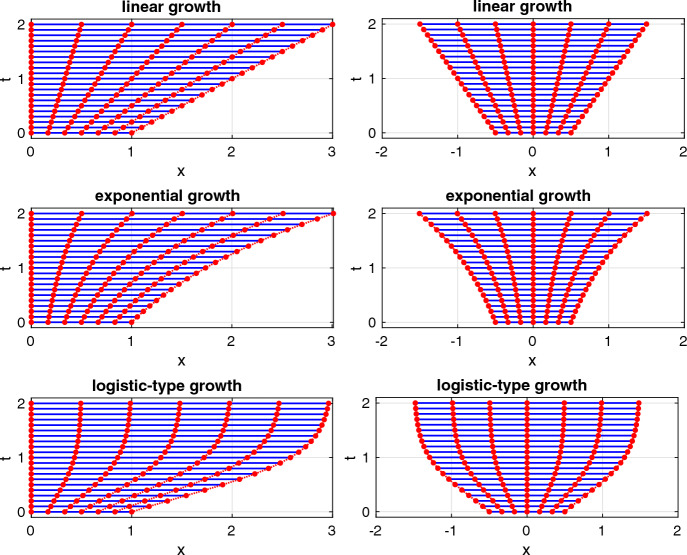
Illustrations of a homogeneous domain change in growing Type L (left column) and Type S (right column) domains. In each plot, the spatial domains $\Omega _t$ at a set of time points *t* are marked as blue lines; the red dotted lines and the red dots give the trajectories of the uniformly spaced grid points on the domain $\Omega _0$. The growth is linear (top row), exponential (middle row), and of logistic-type (bottom row). Parameters are: $t_0=0$, $T= 2$, $L_0=1$, $\alpha =1$ in linear and $\alpha =0.55$ in exponential case, $\beta =2.5$, $L_\infty =3$

**Fig. 6:**
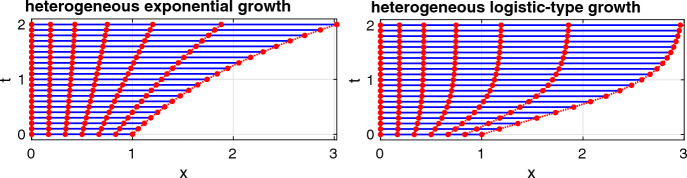
Illustrations of a heterogeneous domain change defined by $\partial _x v(t,x)=r(t)x$ in growing Type N domains. The left plot shows an exponential and the right plot a logistic-type growth of the overall domain. Parameters are as in Fig. 5

## Data Availability

Data sharing is not applicable to this article as no new data were created or analysed in this study. The software used in the preparation of this article is available at https://git-ce.rwth-aachen.de/AGpub/Gerisch2026-BullMathBiol

## Spatially One-Dimensional Time-Dependent Domains

In Table 1 we give the expressions for *L*(*t*) and its derivative $\dot{L}(t)$ which correspond to linear, exponential, and logistic-type change in domain length. In all three cases, the parameter $L_0>0$ gives the length at initial time, $L(t_0)=L_0$, and, in the first two cases, parameter $\alpha$ indicates a growing domain for $\alpha >0$ and a shrinking domain for $\alpha <0$. In the logistic-type case, $L_\infty$ is the limiting domain size for $t\rightarrow +\infty$ and $\beta >0$ the rate at which this limit is approached; obviously, here the domain is growing for $L_\infty >L_0$ and shrinking for $L_\infty <L_0$.

**Table 1:** Expressions for one-dimensional domains with linear, exponential, and logistic-type length change. The parameters are described in the text

type of change	*L*(*t*)	$\dot{L}(t)$
linear	$L_0+\alpha (t-t_0)$	$\alpha$
exponential	$L_0\exp (\alpha (t-t_0))$	$\alpha L(t)$
logistic-type	$\frac{L_0L_\infty }{L_0+(L_\infty -L_0)\exp (-\beta (t-t_0))}$	$\frac{L(t)\beta (L_\infty -L_0)\exp (-\beta (t-t_0))}{L_0+(L_\infty -L_0)\exp (-\beta (t-t_0))}$

These domains are illustrated in Fig. 5 for the case of a homogeneous domain change in growing Type L and Type S domains. In order to illustrate the nature of the homogeneous growth of the domains, we also plot the trajectories of grid points of a uniform grid on the initial domain $\Omega _{t_0}$ over time. The grid at later times remains uniform here, although with increasing grid spacing.

We can achieve the same overall domain length *L*(*t*) over time but with heterogeneous domain change. Here we follow an approach suggested in Simpson et al. (2006) and make the local rate of change in domain length dependent on *x* by letting $\partial _x v(t,x)=r(t)x$ . Inserting in (5) gives

$$ \dot{L}(t) = \int _0^{L(t)}r(t)(l(t)+x)\,\textsf{d}{x} = r(t)L(t)\left( l(t)+\frac{L(t)}{2}\right) \,. $$

Assuming $l(t)+L(t)/2\ne 0$, which rules out Type S domains, this gives an expression of *r*(*t*) in terms of *L*(*t*), its derivative, and *l*(*t*). Integrating the special choice of $\partial _x v(t,x)$ gives $v(t,x) = r(t)x^2/2+C(t)$. As in the homogeneous case, we determine *C*(*t*) from (1) with starting value $l(t_0)$ which gives $\dot{l}(t)=v(t,l(t))$, hence $C(t)=\dot{l}(t)-r(t)l(t)^2/2$ and thus

$$ v(t,x) = \frac{r(t)}{2}\left( x^2-l(t)^2\right) +\dot{l}(t)\,. $$

For Type L domains, i.e. $l\equiv 0$, this simplifies to

$$ r(t) = \frac{2\dot{L}(t)}{L(t)^2} \quad \text {and}\quad v(t,x) = \frac{r(t)x^2}{2} = \frac{\dot{L}(t) x^2}{L(t)^2}\,. $$

The resulting Type L domains for exponential and logistic-type domain length change are shown in Fig. 6. Observe the non-uniform grid arising with time and, due to the chosen $\partial _x v(t,x)=r(t)x$, that the domain is growing faster at its expanding end but the overall domain length change is as in the corresponding homogeneous case.

## Fourier-Type Stability Analysis for the Aggregating Cellular Population Model

Here we consider the aggregating cellular population model, see §5.1, on a *fixed spatial domain* (0, *L*) *with domain length* *L* and with periodic boundary conditions. We are interested in the stability with respect to spatial perturbations of homogeneous steady state solutions of this model. We investigate this with a Fourier-type stability analysis. This analysis follows that given in (Armstrong et al., 2006, Section 3.2) but there the spatial domain is the full real line, *i.e.* no boundary, and not a bounded domain of length *L*. We are interested under which conditions on model parameters, including the domain length *L*, the homogeneous steady state is unstable and patterns can arise.

The starting point for this investigation is thus Eq. (22) with $v\equiv 0$ (fixed domain) and $f_{\text {(p)}}\equiv 0$ (no proliferation) together with the non-local interaction term (18) and the interaction function (19) where we use, as throughout this work, the simple form $g(u):=u$. Thus for $x\in (0,L)$ and $t\in (t_0,T]$ we consider

B1 $$\begin{aligned} \partial _t u =-\partial _x\left( -D\partial _x u+up(u) \frac{\omega \mu }{\xi }\int _{-\xi }^\xi u(t,x+r)\frac{r}{|r|}\tilde{\Omega }(|r|/\xi )\,\textsf{d}{r} \right) \end{aligned}$$

together with periodic boundary conditions and an initial condition at time $t_0$.

Note that in (B1) we have the volume filling term *p*(*u*) in front of the integral while that term appears as an additional factor of the integrand in Armstrong et al. (2006). Since we aim to study the onset of aggregation from a perturbed spatially homogeneous steady state well below the limiting packing density *P*, we will consider below the case $p(u)\equiv 1$ and this difference disappears.

The first step is to non-dimensionalise (B1) and we define

$$ x^*=\frac{x}{\tilde{x}}\,,\quad t^*=\frac{t}{\tilde{t}}\,,\quad u^*=\frac{u}{\tilde{u}}\,,\quad \text {where } \tilde{x}:=\xi \,,\quad \tilde{t}:=\frac{\tilde{x}^2}{D}\,,\quad \tilde{u}:=\frac{D}{\omega \xi }\,. $$

This gives in the case $p(u)\equiv 1$ and on the spatial domain $(0,L^*)$, where $L^*:=L/\xi$,

B2 $$\begin{aligned} \partial _{t^*} u^*=\partial _{x^*}^2 u^*-\partial _{x^*}\left( u^*\mu \int _{-1}^1 u^*(t^*,x^*+r^*)\frac{r^*}{|r^*|}\tilde{\Omega }(|r^*|)\,\textsf{d}{r^*} \right) \,. \end{aligned}$$

Next we observe that $u^*\equiv U^*$ is a homogeneous steady state of (B2) for any $U^*>0$ because for such $u^*$ the integrand in (B2) becomes an odd function and the integral vanishes. Consider a perturbed initial condition $U^*+\bar{u}(t^*_0,x^*)$ with small perturbation $\bar{u}(t^*_0,x^*)$. We linearise (B2) around $U^*$ by replacing $u^*(t^*,x^*)$ with $U^*+ \bar{u}(t^*,x^*)$ and dropping nonlinear terms in $\bar{u}$. This results in the following linear equation for $\bar{u}$

B3 $$\begin{aligned} \partial _{t^*} \bar{u} =\partial _{x^*}^2 \bar{u}-\partial _{x^*}\left( U^*\mu \int _{-1}^1 \bar{u}(t^*,x^*+r^*)\frac{r^*}{|r^*|}\tilde{\Omega }(|r^*|)\,\textsf{d}{r^*} \right) \end{aligned}$$

on the spatial domain $(0,L^*)$ and $\bar{u}$ fulfils periodic boundary conditions.

Due to this periodicity, Fourier modes for $\bar{u}$ take the form $\textrm{e}^{\lambda _k t^*}\textrm{e}^{i \frac{2\pi k}{L^*}x^*}$ with integer wave number *k* and corresponding temporal eigenvalue $\lambda _k$. For a shorter notation we define $k^*:=2\pi k / L^*$. Substituting such a Fourier mode as solution $\bar{u}$ into (B3), we obtain as expression for the temporal eigenvalues, when using the normalised interaction function $\tilde{\Omega }$ as in Eq. (20),

B4 $$\begin{aligned} \lambda _k = -(k^*)^2 -i \mu U^*k^*\int _{-1}^1 \textrm{e}^{i k^*r^*} \frac{r^*}{|r^*|}\tilde{\Omega }(|r^*|)\,\textsf{d}{r^*} = -(k^*)^2 -2 \mu U^*\left( \cos (k^*)-1\right) \,. \end{aligned}$$

A Fourier mode with wave number $k>0$ (these are the non-constant Fourier modes) is amplified in time if the corresponding temporal eigenvalue $\lambda _k$ has positive real part. Consequently, if such $k>0$ exist, the corresponding perturbations of $U^*$ are amplified and patterns can emerge. The Fourier mode with the largest (positive) temporal eigenvalue is called the dominant mode and it dictates the (initial) pattern since it is amplified the strongest.

We observe from (B4) that $\lambda _k = \lambda _{-k}\in \mathbb {R}$. Consequently, if the Fourier mode with wave number *k* is amplified then also the one with wave number $-k$, and we only look at the eigenvalues $\lambda _k$ corresponding to wave numbers $k=1,2,\dots$ in the following.

The expression (B4) for the $\lambda _k$ here resembles the corresponding expression in Armstrong et al. (2006) except that the $k^*$ here is simply the wave number *k* there. Consequently, as in that paper, we conclude that for sufficiently large interaction strength $\mu \ge \mu _\text {min}$ there will be wave numbers $k>0$ with $\lambda _k>0$ and pattern formation (aggregation of cells) takes place.

In contrast to Armstrong et al. (2006), we here consider a fixed bounded domain with periodic boundary conditions. Consequently, the domain length *L* is another parameter and it has an influence on which Fourier modes are amplified and which of them is the dominant mode. Also the threshold interaction strength $\mu _\text {min}$ may depend on *L*. We consider the wave number $k>0$ as a continuous parameter $k\in \mathbb {R}$ for the moment and denote the temporal eigenvalues for a given $L>0$ by $\lambda _k(L)$. The temporal eigenvalues of different domain lengths *L* and $\phi L$ with $\phi >0$ are related and it holds

$$ \lambda _{k}(\phi L) = \lambda _{k/\phi }(L)\quad \text {for all }k\,, \phi > 0\,. $$

We consider two cases (one the logical negation of the other).

1. *For some* $\mu >0$ *and given* $L>0$ *holds* $\lambda _{k}(L)\le 0$ *for all real* $k>0$. Then, for that $\mu$ and all real $k>0$, we have $\lambda _{k}(\phi L)\le 0$ for any $\phi >0$. This holds in particular for all integer $k>0$. Thus no pattern can emerge for that $\mu$ independent of the domain size *L*.
2. *For some* $\mu >0$ *and given* $L>0$ *there exist real* $k>0$ *such that* $\lambda _{k}(L)> 0$ In this case there exist (one or more) intervals for *k* on the positive real line, we refer to them as the *k**-intervals for domain length* *L* below, such that $\lambda _{k}(L)> 0$ on these intervals. Then, for that $\mu$, we have the existence of corresponding *k*-intervals for domain length $\phi L$ such that $\lambda _{k}(\phi L)> 0$ on them for any $\phi >0$. Note that if (*a*, *b*) is any *k*-interval for *L* then the corresponding *k*-interval for $\phi L$ is $(\phi a, \phi b)$. Thus, in comparison to the *k*-intervals for domain length *L*, the corresponding *k*-intervals for domain length $\phi L$ move to the right and increase in length for increasing domain length ($\phi >1$) and move to the left (but remain to the right of zero) and decrease in length for decreasing domain length ($0<\phi <1$). From the fact that for increasing domain length *L* the corresponding *k*-intervals move to the right and increase in length, we can expect that the dominant wave number also increases with increasing *L*.

The transition to integer values $k>0$ is not straightforward in this second case.

- The *k*-intervals for domain length *L* may (or may not) contain integer values *k* and consequently patterns do (or do not) emerge on domains of length *L*.
- Regardless of what is the case for domains of length *L*, the corresponding *k*-intervals for domain length $\phi L$ may (or may not) contain integer values *k* and consequently patterns do (or do not) emerge on domains of length $\phi L$.
- However, for sufficiently large $\phi \ge 1$ at least one of the corresponding *k*-intervals for domain length $\phi L$ will have length one at least and thus contain an integer value. Hence patterns do emerge on domains of length $\phi L$ and consequently also on all domains of length larger than $\phi L$. Thus patterns emerge for that $\mu$ for sufficiently large domain size *L*.

To illustrate the discussion above, we look at the particular parameter values used in the simulations presented in §5.2. In Table 2 we give the values of $\mu _\text {min}$ for a wide range of fixed domain lengths *L*. From this we infer that the dependence of $\mu _\text {min}$ on the domain length *L* is weak.

**Table 2:** The bottom row shows the values of $\mu _\text {min}$ for the aggregating cellular population model on the periodic domain of fixed length *L* (top row). Model parameters are as in (25)

length *L*	2.0	2.5	3.0	5.4	7.5	10.0	100.0	1000.0
$\mu _\text {min}$	0.0110	0.0105	0.0100	0.0100	0.0095	0.0095	0.0095	0.0095

Furthermore, taking the parameter values used in the simulation shown in Fig. 2 and considering the range of domain lengths of the time-dependent domain there over the time period $t\in [0,10]$, we obtain, for the corresponding fixed domains, the dominant modes (wave numbers *k*) as given in Table 3. As expected, we read from that table that for increasing domain length also the dominant wave number increases.

**Table 3:** The dominant wave number *k* (bottom row) of the aggregating cellular population model on the periodic domain of fixed length *L* (middle row). The given domain lengths *L* are attained at the time points *t* (top row) in the simulation of the model on the time-dependent domain as shown in Fig. 2. All other model parameters are also as used in that simulation.

time *t*	0	1	2	3	4	5	6	7	8	9	10
length *L*	2.00	2.21	2.44	2.70	2.98	3.30	3.64	4.03	4.45	4.92	5.44
dominant *k*	2	3	3	3	4	4	5	5	6	6	7

## Footnotes

1 Indirect contacts, as for instance mediated by a chemotactic chemical, are not considered here.

2 The notation $\Omega (|r|; \xi ,\mu )$ for the interaction function coincides with that used in Painter et al. (2015); it should not be confused with the notations of the space-time domain $\Omega _I$ and of the transformed spatial domain $\hat{\Omega }$ as introduced in §2.
